# An Allosteric-Probe for Detection of Alkaline Phosphatase Activity and Its Application in Immunoassay

**DOI:** 10.3389/fchem.2018.00618

**Published:** 2018-12-12

**Authors:** Jingjing Guo, Mingxuan Gao, Yanling Song, Li Lin, Kaifeng Zhao, Tian Tian, Dan Liu, Zhi Zhu, Chaoyong James Yang

**Affiliations:** ^1^MOE Key Laboratory of Spectrochemical Analysis and Instrumentation, Key Laboratory for Chemical Biology of Fujian Province, State Key Laboratory of Physical Chemistry of Solid Surfaces, Collaborative Innovation Center of Chemistry for Energy Materials, College of Chemistry and Chemical Engineering, Xiamen University, Xiamen, China; ^2^Institute of Molecular Medicine, Renji Hospital, School of Medicine, Shanghai Jiao Tong University, Shanghai, China

**Keywords:** aptamer, alkaline phosphatase, allosteric-probes, complex sample, immunoassay

## Abstract

A fluorescence strategy for alkaline phosphatase (ALP) assay in complicated samples with high sensitivity and strong stability is developed based on an allosteric probe (AP). This probe consists of two DNA strands, a streptavidin (SA) aptamer labeled by fluorophore and its totally complementary DNA (cDNA) with a phosphate group on the 5′ end. Upon ALP introduction, the phosphate group on the cDNA is hydrolyzed, leaving the unhydrolyzed cDNA sequence for lambda exonuclease (λ exo) digestion and releasing SA aptamer for binding to SA beads, which results in fluorescence enhancement of SA beads that can be detected by flow cytometry or microscopy. We have achieved a detection limit of 0.012 U/mL with a detection range of 0.02~0.15 U/mL in buffer and human serum. These figures of merit are better than or comparable to those of other methods. Because the fluorescence signal is localized on the beads, they can be separated to remove fluorescence background from complicated biological systems. Notably, the new strategy not only applies to ALP detection with simple design, easy operation, high sensitivity, and good compatibility in complex solution, but also can be utilized in ALP-linked immunosorbent assays for the detection of a wide range of targets.

## Introduction

Alkaline phosphatase (ALP), a type of hydrolase enzyme, is widespread in nature for removing phosphate groups from various biomolecules like nucleic acids, proteins, and carbohydrates. ALP is present in all tissues in the human body. Due to ALP's important role in metabolism in the liver and development of the skeleton, an abnormal level of ALP is often related to bone disease (Garnero and Delmas, 1993), liver dysfunction (Ooi et al., 2007), diabetes (Berberoglu et al., 2007), and breast cancer (Brar et al., 1993). Hence, development of ALP detection methods is important for clinical diagnosis and cancer screening. In addition, like horseradish peroxidase (HRP), ALP can conjugate to streptavidin (Zhou et al., 2016) or a detection antibody for signal amplification and output in enzyme-linked immunosorbent assay (ELISA) (Porstmann et al., 1985). Therefore, development of new assays for ALP not only satisfies clinical diagnostic requirements but also improves the performance of ALP-labeled ELISAs.

Because ALP catalyzes the dephosphorylation processes of various substrates, many methods have been applied to quantify ALP's enzymatic activity, including fluorescence (Liang et al., 2013; Qian et al., 2015; Hu et al., 2016), colorimetry (Jiao et al., 2014; Yang et al., 2016), electrochemistry (Barroso et al., 2016), chemiluminescence (Jiang and Wang, 2012), and surface-enhanced Raman spectroscopy (Ruan et al., 2006). Most assays employ small molecules as substrates, including pyrophosphate (PPi), ascorbic acid 2–phosphate (AAP), p-nitrophenyl phosphate (PNPP), and p-aminophenyl phosphate (PAPP). Due to the easy labeling with a signal molecule and digestion by nuclease, nucleic acids are ideal phosphorylation substrates. Lambda exonuclease (λ exo) is a highly processive 5'-3' double-stranded DNA (dsDNA) exodeoxyribonuclease, which can selectively digest the 5'-phosphorylated strand of dsDNA, while work inefficiently on single-stranded DNA (ssDNA) and non-phosphorylated DNA. Miao et al. reported an electrochemical strategy to detect ALP by using dsDNA probes, and λ exo was employed for enzyme-mediated signal amplification (Miao et al., 2011). However, the complex preparation steps for electrochemical sensors make the workload heavier and test time longer. In addition, the poor limit of detection (LOD) (100 U/L) still needs improvement. Colorimetric strategies exploiting nucleic acids as the substrates coordinating with λ exo for ALP detection have also been developed. For example, a perylene probe can induce the aggregation of gold nanoparticles, which causes a color change from pink red to dark blue (Jiao et al., 2014). Disadvantages include preparation difficulty and poor LOD (32 U/L), limiting the practical applications in biological samples. More recently, Liu et al. developed a new fluorescent ALP assay using prior binding of graphene oxide (GO) to ssDNA over dsDNA (Liu et al., 2016), resulting in a lower LOD (0.19 U/L), but performance of this method may be compromised in human serum without a separation step. Overall, it is apparent that these sensors often suffer from complicated probe preparation and detrimental effects of the biological matrix, such as the influence of sample autofluorescence. So far, the detection of ALP in complicated samples in these methods is mostly performed in 1% human serum (Ma et al., 2016) or cell extracts (Jiao et al., 2014). Higher concentrations of complicated samples may affect the probes, leading to failure of the biological assay. To solve these problems, new signaling strategies with high sensitivity and strong stability in complicated samples are highly desired for ALP detection.

In our previous work, we proposed the design and application of allosteric molecular beacons (aMBs) (Song et al., 2011, 2012; Zhang et al., 2016; Gao et al., 2017) for highly sensitive and selective detection of nucleic acids, proteins and small molecules in complicated biological systems. Briefly, an aMB is an ssDNA labeled by fluorophore containing a molecular recognition sequence, streptavidin (SA) aptamer and its cDNA. The hybridization allows the probe to form a stable hairpin structure, preventing the probe from binding to SA beads. When the target is present, the recognition sequence binds to the target, which opens the hairpin structure to release SA aptamer, allowing the probe to bind to SA beads, consequently leading to fluorescent signal readout. The bead-based separation (Huang and Liu, 2010) and the single-fluorophore-labeled reporter contribute to the low background and little influence of matrix effects.

Herein, we report the design of an allosteric probe (AP) based on an aMB to improve the ALP detection performance in complex environments. The AP contains two complementary sequences, an SA aptamer labeled by fluorophore and its total cDNA modified with a 5'-phosphoryl terminus. In the absence of ALP, the cDNA is promptly cleaved by λ exo to release the SA aptamer, which binds to SA beads within 5 min incubation, resulting in fluorescence enhancement of SA beads that can be detected by microscopy or flow cytometry. In contrast, ALP catalyzes hydrolysis of the 5'-phosphoryl terminus from the cDNA, producing a 5'-hydroxyl end product that prevents λ exo cleavage. As a consequence, no SA aptamers bind to SA beads yielding weak fluorescence. Compared to the reported ALP detection methods, interfering autofluorescence from species in the complex biological system can be easily isolated by a bead-based separation, such as simple centrifugation or filtration, to reduce non-specific adsorption-induced background. Besides, a large surface-to-volume ratio enriches the fluorescent signals on the beads which improves the signal-background-ratio (SBR). Without the need for nucleic acid amplification, the developed AP avoids the use of multiple enzymes and consequent complicated operation and cross-contamination, thereby decreasing operational difficulty and time-consumption for signal readout. The results show a detection limit of 0.012 U/mL and a linear response over the range of 0.02–0.15 U/mL in buffer and human serum with high sensitivity and selectivity. Furthermore, the method can be applied to the inhibitor screening of ALP and ALP-labeled ELISA, expanding the scope of practical applications. This strategy introduces a new way to detect ALP and shows the potential for applications in drug discovery and cancer diagnosis.

## Materials and Methods

### Materials

Alkaline phosphatase (ALP, 1000 U/mL) and p-nitrophenyl phosphate (PNPP) were purchased from Thermo Fisher Scientific Inc (Shanghai, China). Lambda exonuclease (λ exo, 5000 U/mL) and T4 ligase were obtained from New England Biolabs (Ipswich, MA, USA). Thrombin was supplied from Haematologic Technologies (Essex Junction, VT, USA). Lysozyme was purchased from R&D Systems (Minneapolis, MN, USA). Human serum albumin (HSA) was bought from Tagene Biotechnology Co., Ltd. (Xiamen, China). Proteinase K was purchased from BBI Science (Shanghai, China). Trypsin was purchased from Biofroxx (Einhausen, Germany). Na_3_VO_4_, human IgG and anti-human IgG-coated ELISA plates were purchased from Sigma-Aldrich (St. Louis, MO, USA). L-cysteine was bought from Sinopharm Chemical Reagent Co. Ltd (Shanghai, China). Human serum was obtained from Affiliated Chenggong Hospital (Xiamen University). Streptavidin (SA) Sepharose beads were purchased from GE Healthcare (Chicago, IL, USA). ALP-labeled goat anti-human IgG was purchased from Abcam (Cambridge, MA, USA). RIPA and PMSF were bought from Solarbio life sciences (Beijing, China). All other chemicals were analytical reagent grade and used without further purification. All oligonucleotides used in this study were synthesized and HPLC purified by Sangon Biotechnology Co., Ltd. (Shanghai, China) and sequences are listed in Table S1.

### Optimization of the AP

To obtain the best SBR, the SA aptamer sequences were optimized. To prepare the DNA for hybridization, 10 μM AP (including the SA aptamer and its total cDNA) was heated to 95°C for 5 min and then held at 37°C for 30 min. In a typical ALP assay, 200 nM AP in 100 μL buffer (70 mM Tris-HCl, 10 mM MgCl_2_, pH = 8.0) with 10 U/mL ALP was incubated at 37°C for 10 min. Then 100 U/mL λ exo was added to the solution and incubated at 37°C for 30 min to digest the 5'-phosphorylated cDNA. After incubation, the enzyme was inactivated at 75°C for 10 min. The mixture was cooled to room temperature, and 3 μL SA beads was added and rotated slowly for 5 min to interact with the free SA aptamers. After washing 3 times with buffer, the fluorescence of SA beads was quantitatively characterized by flow cytometry or fluorescence microscopy.

### Quantitative Assay of ALP Activity

To achieve quantitative detection of ALP activity, a series of different concentrations of ALP was added to the reaction system. For complex biological systems, 10% human serum was utilized as the reaction buffer with different concentrations of ALP. After the enzyme-deactivation step, the precipitate was removed by centrifugation. The subsequent steps were the same as those described in the previous section.

### Selectivity Study

The selectivity of the ALP assay was investigated by using other enzymes or proteins, including thrombin, T4 ligase, lysozyme, HSA, proteinase K, trypsin and de-activated ALP. The concentration of ALP was 2 U/mL, and the concentrations of other enzymes and proteins were 20 U/mL or 10 nM. Other procedures were the same as those stated above.

### ALP Inhibitor Screening

The ALP inhibitor screening was performed by utilizing Na_3_VO_4_ and L-cysteine. Different concentrations of inhibitors were mixed with 2 U/mL ALP, and the mixture was added to 200 nM AP in 100 μL buffer. The remaining steps were the same as those stated above.

### ALP-Linked Immunosorbent Assay

To achieve quantitative detection for human IgG activity, each well of an anti-human IgG coated ELISA plate was filled with human IgG (100 μL, 30–960 ng/mL) at ambient temperature for 2.5 h with gentle shaking and then washed 4 times with washing buffer (300 μL). Detection antibody, ALP-labeled goat anti-human IgG (100 μL, 1:1000) was added to each well, and incubated for 1 h at room temperature with gentle shaking and then washed 4 times with washing buffer (300 μL). Then, 200 nM AP in 100 μL buffer was added to each well and incubated at 37°C for 10 min. After incubation, all the solutions were transferred to centrifuge tubes, and the remaining steps were the same as those stated above.

### Preparation of Cell Lysates

Hela cells were cultured in DMEM medium with 10% fetal bovine serum (FBS). The cells were washed three times with PBS, and treated with 1 mL RIPA and 10 μL PMSF for 1 h on ice. A suspension of 10^5^ cells/mL was centrifuged at 10,000 rpm for 30 min at 4°C, and the supernatant was used as complex matrix and spiked with ALP for detection.

### ALP Detection Using PNPP Substrate

Four microgram per microliter PNPP substrate dispersed in diethanolamine was prepared and added to 96-well plate. A series concentration of ALP was added to each well and incubated for 30 min at 37°C. Then the stop solution of 2 M NaOH was added for stopping reaction. Finally, the absorbance of 405 nm was recorded by microplate reader.

## Results and Discussion

### Working Principle

**Figure 1:**
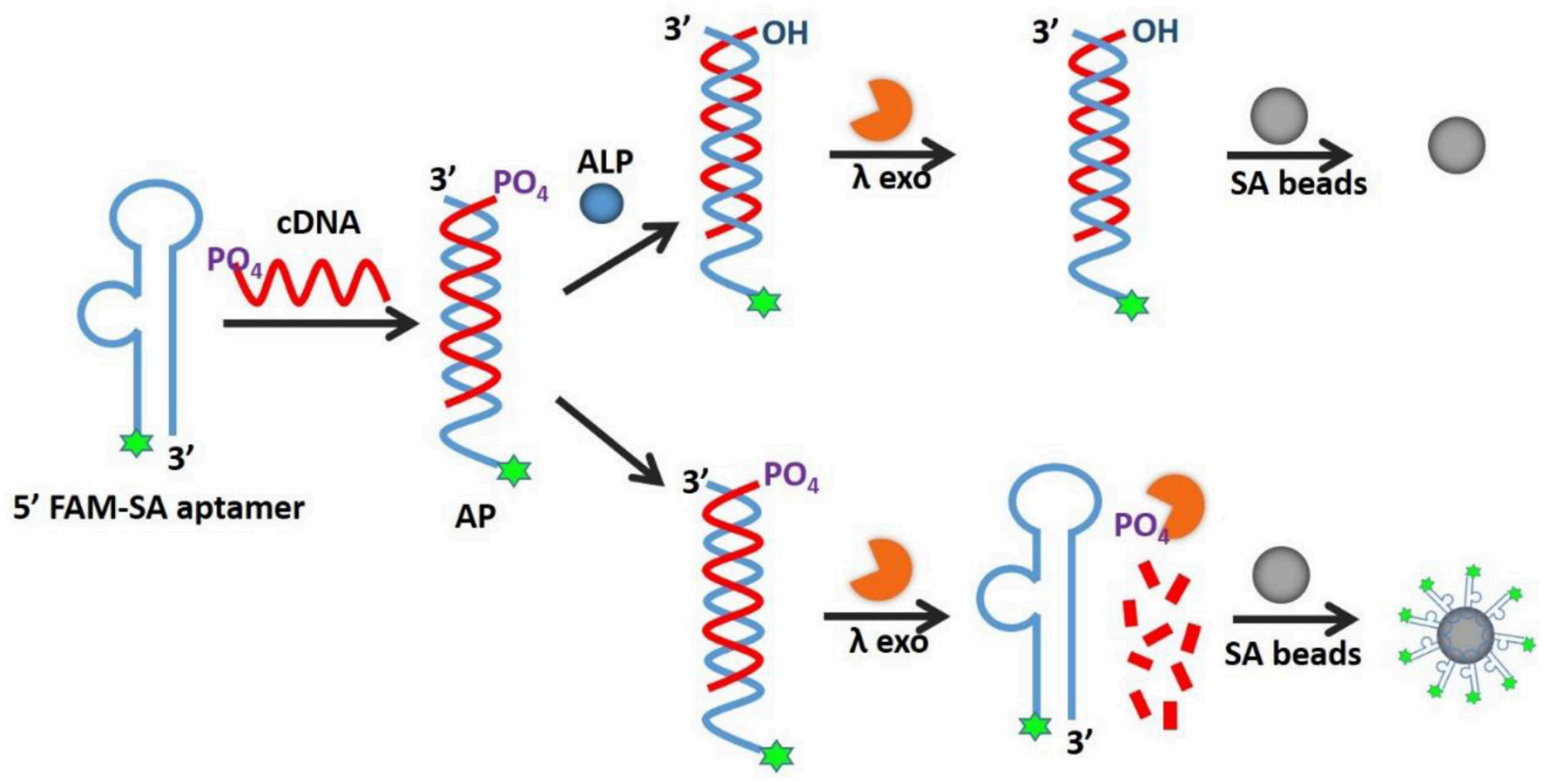
Schematic illustration of AP for detection of alkaline phosphatase activity.

As illustrated in Figure 1, the AP holds its duplex conformation with ALP and changes to single-stranded form without ALP, which means allosteric effect, resulting in the fluorescence readout for the detection. An AP is composed of two complementary sequences, including an SA aptamer labeled by fluorophore and its total cDNA modified with a 5'-phosphoryl terminus. When ALP is present, the 5'-phosphoryl terminus of cDNA would be hydrolyzed, thus avoiding cleavage by λ exo and blocking the SA aptamer binding affinity, resulting in low fluorescence. Without ALP, the terminus of the cDNA would remain phosphorylated, leading to degradation by λ exo to release the fluorophore-labeled SA aptamer for binding to SA beads. The decreased fluorescence of SA beads in the presence of ALP is quantitatively detected by fluorescence microscopy or flow cytometry.

### Optimization of the AP and Reaction Conditions

To achieve highly sensitive detection of ALP, we designed the AP by imitating the allosteric effect in nature and optimized the SA aptamer sequences, ALP incubation time, and the concentrations of lambda exo. The sensitivity of the method relies heavily on the allosteric effect of the AP, which depends on the relative binding affinities of SA aptamer to streptavidin vs. cDNA. As shown in Figure S1 (A), we chose three SA aptamers from the literature (Bing et al., 2010), named SA1, SA2, and SA3, with different compositions of the stem and bulge. We equilibrated SA aptamers and their cDNAs in buffer with or without ALP, then added λ exo and SA beads in succession, and finally calculated the SBR (F_0_/F, where F_0_ and F are fluorescence intensities without and with ALP, respectively). As shown in Figure S1 (B), SA3 provided best SBR among all three aptamers. Since the binding affinities of SA1 and SA2 to streptavidin are higher than that of SA3, when ALP is present, SA1 and SA2 are more inclined to dissociate from duplex sequences and bind to SA beads, resulting in false positive signals which decreased their SBRs. Thus, SA3 was chosen for subsequent experiments.

Furthermore, as shown in Figure S2, we optimized the ALP incubation time from 10 to 60 min, and chose the shortest one of 10 min, since the reaction efficiency is similar for all time durations. Considering that λ exo plays an important role in sequence degradation, we optimized λ exo concentrations including 25, 50, 100, and 150 U/mL. With insufficient λ exo, the degradation efficiency on the 5'-phosphoryl terminus of cDNA3 decreases clearly. While excessive λ exo also causes non-specific degradation and waste of reagents. As shown in Figure S3, 100 U/mL λ exo was chosen for subsequent experiments. We also optimized λ exo incubation time ranging from 15 to 90 min (Figure S4). There was little difference for all tested incubation time. We chose 30 min for further experiments just to be sure the complete reaction. Fluorescence enhancement is also affected by the amount of beads present, thus we optimized the SA beads volume with 0.5–5 μL for binding to 200 nM SA3. As shown in Figure S5, 0.5 and 1 μL SA beads exhibited higher fluorescence intensity, but they are easy to lose during washing steps. For 3 and 5 μL SA beads, it seems that fluorescence intensity is a little lower but still noticeable. Finally, 3 μL beads were chosen as the optimal beads volume for further experiments.

### Feasibility of the AP

**Figure 2:**
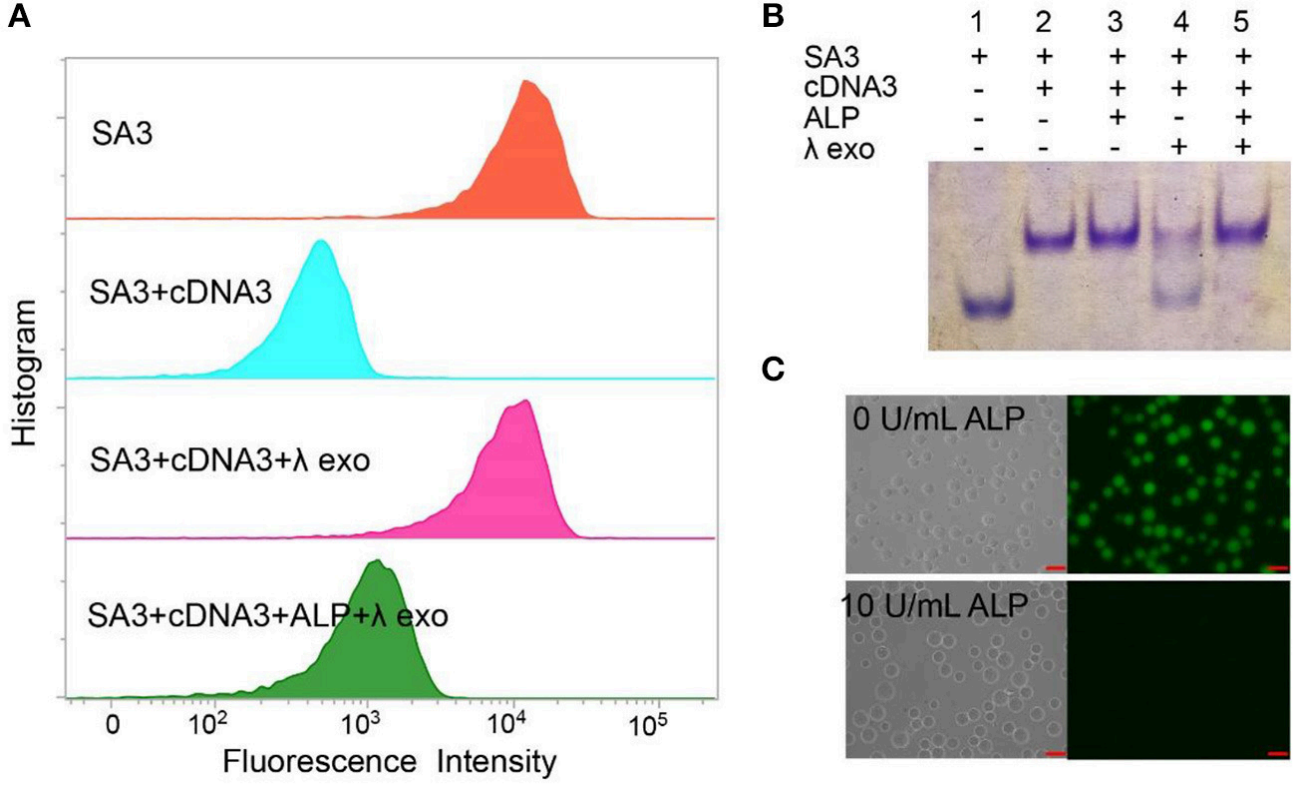
Different methods for proving the feasibility of AP with 10 U/mL ALP: **(A)** flow cytometry, **(B)** 15% native PAGE. Lane 1, SA3; Lane 2, SA3, and cDNA3; Lane 3, SA3, cDNA3, and ALP; Lane 4, SA3, cDNA3, and λ exo; Lane 5, SA3, cDNA3, ALP, and λ exo. **(C)** fluorescence microscope.

We designed the AP consisting of SA3 and cDNA3 to test the feasibility for ALP activity detection. As shown in Figure 2A, the blue peak showed a low fluorescent signal, demonstrating the successful formation of the double-stranded structure to inhibit the aptamer's binding affinity. The pink peak had a higher fluorescence intensity compared to the blue peak, confirming the cleavage of cDNA3 by λ exo. Simultaneously, the fluorescence of the green peak was much lower than that of the pink one, indicating the hydroxylation of the AP by ALP. We also utilized 15% native PAGE to prove the feasibility (Figure 2B). Lane 1 was SA3 for comparison. For lane 2 and 3, in the absence of λ exo, SA3, and cDNA3 remained good hybridization with or without ALP. For lane 4, without ALP, cDNA3 was degraded so that the upper band was decreased as well as SA3 band recovered. For lane 5, ALP dephosphorylated the AP and protected it from λ exo cleavage, leaving the AP intact. Therefore, gel electrophoresis verifies the good hybridization between SA3 and cDNA3, dephosphorylation of 5'-phosphoryl terminus by ALP and specific digestion of phosphorylated ssDNA by λ exo. Furthermore, with the fluorescence microscope (Figure 2C), there was no fluorescence of the AP in the presence of 10 U/mL ALP, while the beads remained highly fluorescent in the absence of ALP. Therefore, the consistent results of flow cytometry, gel electrophoresis, and fluorescence microscopy indicate the feasibility of the AP design.

### Quantitative Measurement of ALP in Buffer and Biological Samples

**Figure 3:**
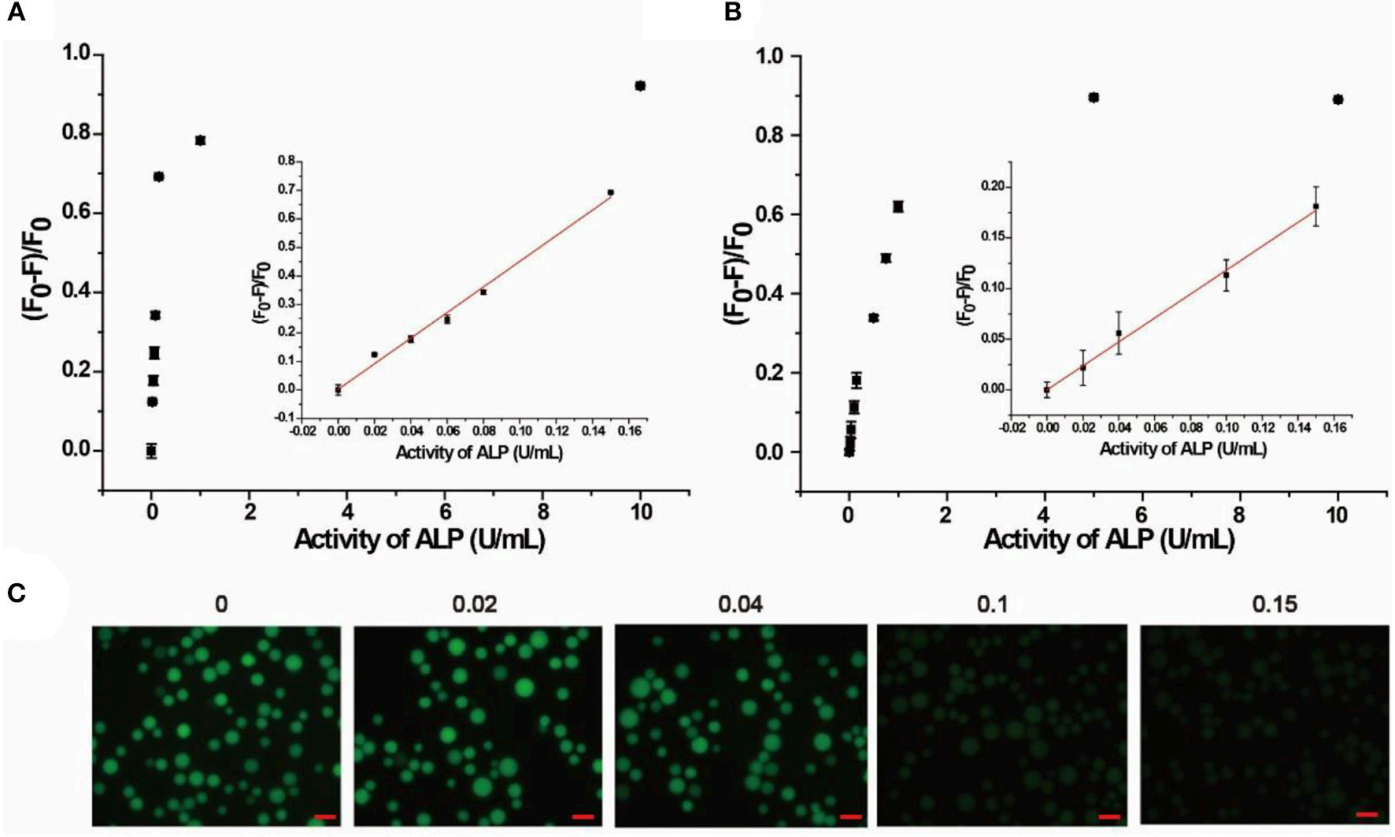
Response of quantitative detection of ALP range from 0 to 10 U/mL in **(A)** buffer solution, **(B)** in 10% human serum monitored by flow cytometry. Inset is the calibration curve ranging from 0.02 to 0.15 U/mL. **(C)** Response of the AP to different concentrations of ALP in buffer solution monitored by fluorescence microscope. Error bars indicate the standard deviations of three samples. Scale bars are 50 μm.

The quantitative detection of ALP was conducted with a series of concentrations of ALP by flow cytometry. As shown in Figure 3A and Figure S6, (F_0_-F)/F_0_ increased with the growing concentration of ALP in buffer, where F_0_ and F are fluorescence intensities without and with ALP, respectively. From the fitting curve in Figure 3A, the LOD was 0.012 U/mL (3σ/S) and the linear range was 0.02–0.15 U/mL (*R*^2^ = 0.9902), which are both superior or comparable to those of other reported methods (Table S2) (Dong et al., 2015; Xing et al., 2016). For clinical applications, the normal range of ALP in human serum is from 0.04 to 0.11 U/mL. In this case, the proposed method covers the normal detection range of ALP in clinical applications and meets the need for clinical diagnosis (Wolf, 1978). As further evidence of the effect of added ALP, fluorescence microscopy was also used for ALP screening. As shown in Figure 3C, distinct fluorescence decrease was observed with an incremental introduction of ALP from 0.02 to 0.15 U/mL, which is consistent with the result of flow cytometry. Therefore, based on the enrichment of fluorescence on beads with no or low levels of ALP, the signal output of the assay could be achieved by different detection methods, including flow cytometry and fluorescence microscope, providing multiple choices for ALP activity screening.

The localization of SA3 on the microbeads made it possible to separate signal generation from the interfering autofluorescence from species in the complicated biological matrix, making the AP applicable to biological samples. To verify this advantage, 10% human serum was chosen as a model complex system for our assay. Since human serum contains multiple components with autofluorescence, including amino acids, vitamins, lipids, and nucleic acid derivatives, as well as growth factor, which added to the background signal and increased homogeneous detection difficulty (Wolfbeis and Leiner, 1985). In our experiment, bead-based separation integrated the signal collection and interfering species separation, leading to highly sensitive and selective detection in complex samples. As shown in Figure 3B, (F_0_-F)/F_0_ increased with increasing concentrations of ALP in 10% human serum. Based on the fitting curve in Figure 3B, the LOD was found to be 0.019 U/mL (3σ/S) and the linear range was 0.02–0.15 U/mL (*R*^2^ = 0.9768), both of which are comparable to those in buffer. In addition, 20% human serum sample was also tested (Figure S7), and similar results were obtained with LOD of 0.021 U/mL (3σ/S) and the linear range of 0.025–0.15 U/mL. Moreover, the AP was also spiked in Hela cell lysate of 10^5^ cells/mL. As shown in Figure S8, the linear range was obtained as 0.05–0.20 U/mL and the LOD was 0.014 U/mL (3σ/S). Therefore, the result indicates that our proposed method is promising for detection in complex biological matrix and clinical sample.

To compare with traditional methods, we also conducted the ALP detection using PNPP substrate. As shown in Figure S9, the linear range was 0.15–0.35 U/mL and the LOD was 0.13 U/mL (3σ/S), which is nearly ten times higher than that of the AP. Therefore, the AP strategy outweighs the traditional method with higher sensitivity.

### Selectivity Study

**Figure 4:**
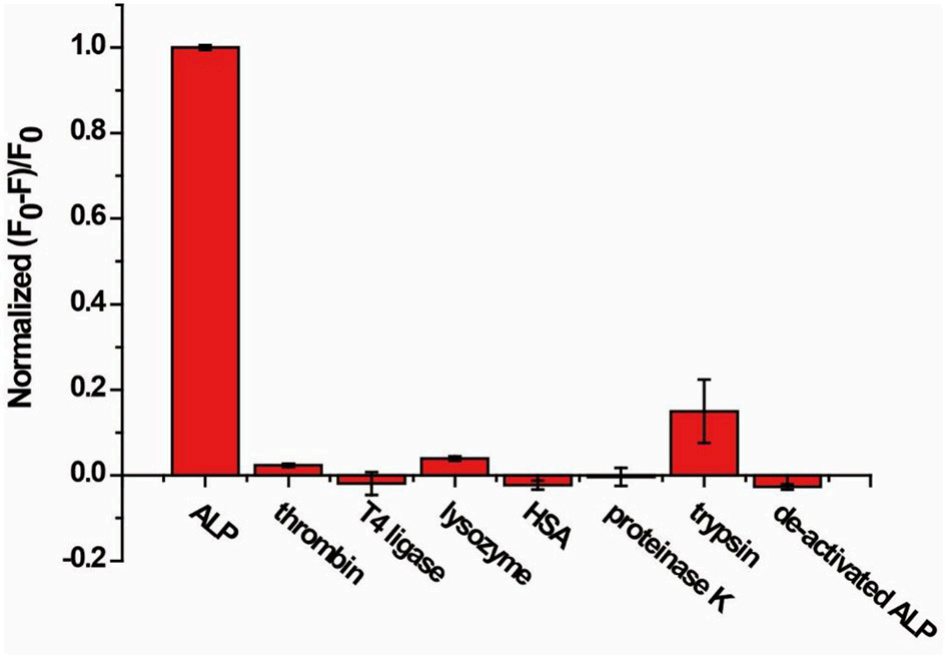
Response of the AP to ALP, thrombin, T4 ligase, lysozyme, HSA, proteinase K, trypsin, and de-activated ALP for specificity study. The concentration of ALP is 2 U/mL, and the concentration of other enzymes and proteins are 20 U/mL or 10 nM. Error bars indicate the standard deviations of three samples.

Selectivity of the AP assay was studied with six non-related enzymes or proteins, including thrombin, T4 ligase, lysozyme, HSA, proteinase K, trypsin, and de-activated ALP. As shown in Figure 4, (F_0_-F)/F_0_ was enhanced only in the presence of ALP, while negligible fluorescence change was observed for other enzymes or proteins with concentrations 10 times that of ALP. The results indicate that our proposed method exhibits good selectivity and enables distinguishing ALP from other interfering species, including other hydrolases such as proteinase K and trypsin.

### Inhibitor Study

As a crucial index for clinical medicine, excess ALP in the human body can induce serious diseases (Hosking et al., 1998). Thus, it is important to study the inhibitor of ALP for medical treatment, drug development and cancer therapy. We chose sodium orthovanadate (Na_3_VO_4_) as a model for ALP inhibitor screening. Sodium orthovanadate is an inorganic compound that exhibits a variety of biological activities, commonly used in inhibition of phosphatases and ATPases for preventing proteins from phosphorylation or promoting phosphorylation of activation (Gordon, 1991). In principle, it is orthovanadate's resemblance to a transition-state analog of phosphate through hydration or chelation that is key to the mechanism of inhibition of ALP by orthovanadate (Seargeant and Stinson, 1979).

**Figure 5:**
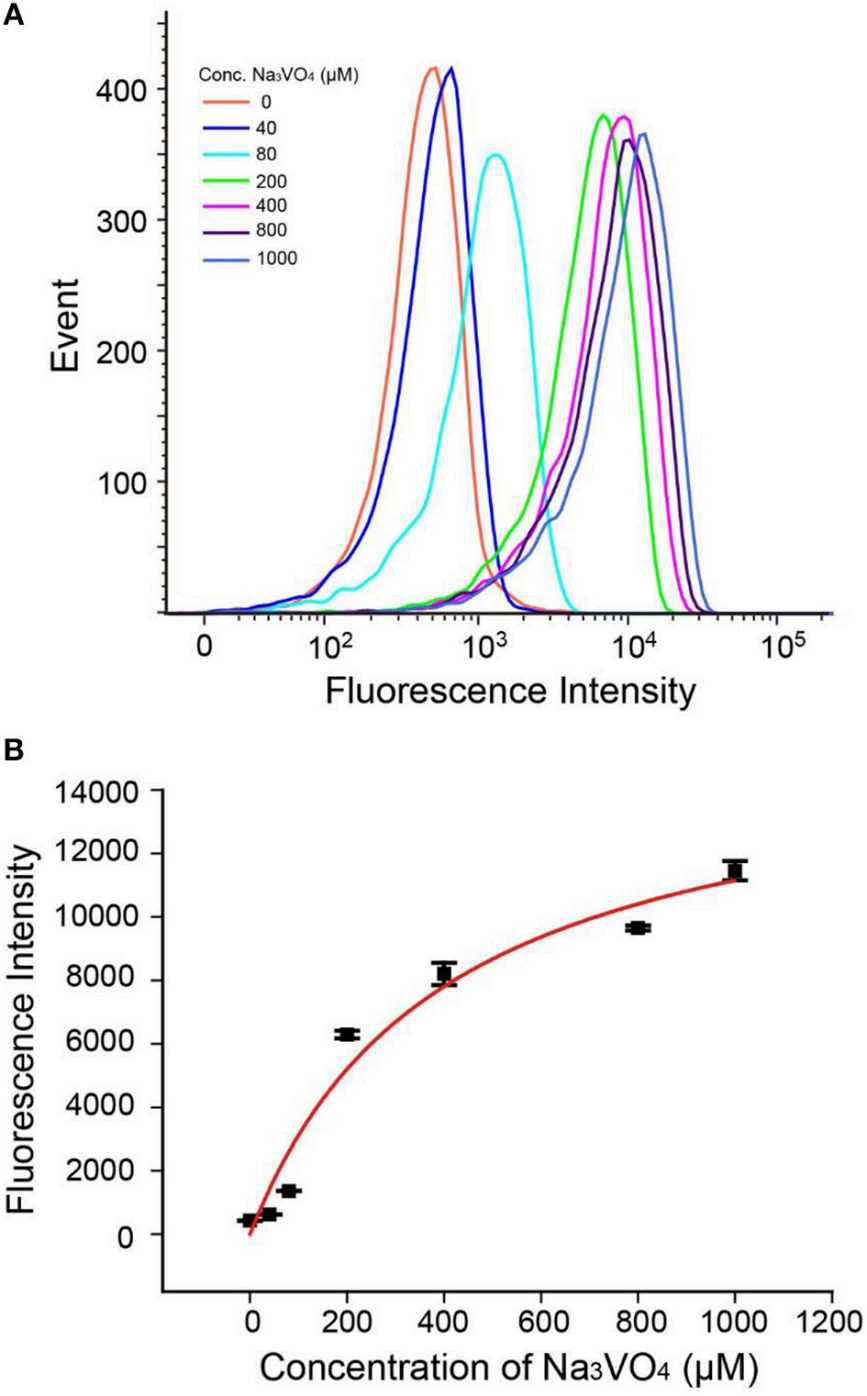
**(A)** Flow cytometry data of Na_3_VO_4_ inhibition efficiency. **(B)** Response of the AP to 2 U/mL ALP with inhibitor Na_3_VO_4_. Error bars indicate the standard deviations of three samples.

As shown in Figures 5A,B, fluorescence intensities of beads read by flow cytometry exhibited gradual enhancement with increasing concentrations of Na_3_VO_4_. The IC50 (half maximal inhibitory concentration) value was found to be 400 μM (±150 μM), which is in good accordance with other reported work (Chen et al., 2014; Wang et al., 2014). Besides, we have studied another inhibitor L-cysteine (Figure S10), the corresponding IC50 was estimated to be 125 μM (±50 μM), which is close to the values obtained by using other methods (Van, 1972), indicating that our method has general applicability in inhibitor screening. Hence, the AP strategy has the potential not only to detect ALP activity, but also to investigate ALP inhibitors and develop new anticancer drugs.

### Quantification of IgG Based on ALP-Linked Immunosorbent Assay

**Figure 6:**
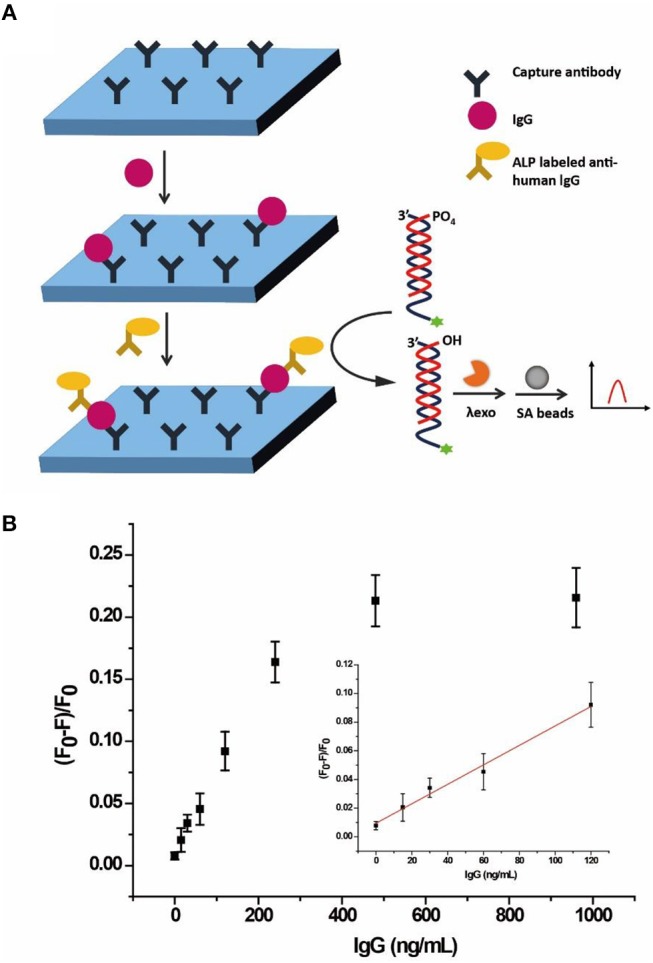
**(A)** Schematic illustration of the allosteric probe for detection of IgG through ALP-labeled immunoassay. **(B)** Response of the AP to different concentrations of human IgG through ALP-linked ELISA in buffer solution monitored by flow cytometry. Error bars indicate the standard deviations of three samples.

Considering its low cost, good stability, high sensitivity, and wide substrate applicability, ALP is generally used to conjugate to streptavidin or a detection antibody for signal amplification in immunoassay (Gao et al., 2014). Since we have demonstrated the feasibility of ALP analysis of the AP, the probe was further applied into an ALP-linked ELISA, taking human IgG as a model. Figure 6A illustrates the integration of the quantification of human IgG through a sandwich immunoassay, ALP enzymatic hydrolysis of the AP and separation of the detection signal on SA beads. In the sandwich immunoassay, capture antibody of anti-human IgG was coated on a 96-well plate. After capture of target human IgG, the detection antibody of ALP-labeled goat anti-human IgG was added to form immune complexes. Then AP was added to react with ALP, resulting in hydrolysis of the 5'-phosphoryl terminus of cDNA3. The λ exo digested the unhydrolyzed AP, releasing the single strand SA3 to be captured by SA beads. As a result, the fluorescence of SA3 was enriched on the beads and monitored by flow cytometry.

As shown in Figure 6B, (F_0_-F)/F_0_ gradually increased with the increasing concentration of human IgG from 0 to 960 ng/mL in buffer. The LOD was 12.9 ng/mL (3σ/S) and the linear range was 15–120 ng/mL (*R*^2^ = 0.9882), where F_0_ and F are fluorescence intensities without and with ALP, respectively. Since the normal range of IgG for healthy adults is 7–16 g/L (Dati et al., 1996), the result clearly showed that the AP with an adequate detection range and sensitivity could be applied to the quantification of human IgG.

## Conclusions

In summary, we have developed a new class of probes based on the allosteric effect, called allosteric probes or AP, which can be applied for ALP activity detection. In AP assay, the single fluorophore-labeled design makes the probe simpler and cheaper than molecular beacons, which requires labeling by both fluorescence and quenching groups. Besides, only one exonuclease is used for cutting DNA sequences, avoiding the need for other assistant enzymes. In addition, the excellent binding affinity and specificity between SA aptamer and SA shortens the binding time to 5 min, and the bead-based enrichment enables a detection limit of 0.012 U/mL without the use of any nucleic acid amplification, especially avoiding complicated manipulations and cross contamination. Furthermore, due to the bead-based separation, the probe can be used in complex biological samples, averting interference from background fluorescence. Moreover, the extending applications of inhibitor study of ALP and analysis of human IgG were also explored. With the advantages of simple design, high sensitivity, easy operation, and good compatibility in complex solutions and in ALP-linked immunosorbent assays, our AP assay has great potential for further applications in ALP inhibitor screening, drug discovery and early diagnosis of cancer. With further research to optimize the probe sequences, the AP could serve as an excellent probe for detection of other kinds of phosphatases and kinases.

## Author Contributions

ZZ, YS, and CY conceived and guided the study. JG performed the experiments and analyzed the data with help from MG, LL, and KZ. JG wrote the first draft of the manuscript. ZZ, YS, DL, and TT contributed to manuscript revision, read, and approved the submitted version.

### Conflict of Interest Statement

The authors declare that the research was conducted in the absence of any commercial or financial relationships that could be construed as a potential conflict of interest.
