# Supplementary material for: An Allosteric-Probe for Detection of Alkaline Phosphatase Activity and Its Application in Immunoassay
Source: Front Chem. 2018 Dec 12;6:618. doi: 10.3389/fchem.2018.00618 (PMC6299030; doi:10.3389/fchem.2018.00618)
Supplement: Supplementary file 1 [file Table_1.docx]

Supplementary Material

An allosteric-probe for detection of alkaline phosphatase activity and its application in immunoassay

Jingjing Guo^1^, Mingxuan Gao^1^, Yanling Song^2*^, Li Lin^1^, Kaifeng Zhao^1^, Tian Tian^1^, Dan Liu^1^, Zhi Zhu^1*^, Chaoyong James Yang^1,2^

^1^ MOE Key Laboratory of Spectrochemical Analysis & Instrumentation, Collaborative Innovation Center of Chemistry for Energy Materials, Key Laboratory for Chemical Biology of Fujian Province, State Key Laboratory of Physical Chemistry of Solid Surfaces, College of Chemistry and Chemical Engineering, Xiamen University, Xiamen 361005 (China)

^2^ Institute of Molecular Medicine, Renji Hospital, School of Medicine, Shanghai Jiao Tong University, Shanghai 200240 (China)

*** Correspondence:** Zhi Zhu, [zhuzhi@xmu.edu.cn](mailto:zhuzhi@xmu.edu.cn);

Yanling Song, [songyanling2012@hotmail.com](mailto:songyanling2012@hotmail.com).

**Table of Contents**

Page

**Table S1.** DNA sequences list. S-3

**Table S2.** The comparison of current methods for ALP detection. S-3

**Figure S1.** The optimization of SA aptamer sequences. S-4

**Figure S2.** The optimization of ALP incubation time in a buffer solution. S-4

**Figure S3**. The optimization of concentrations of lambda exo in a buffer solution. S-5

**Figure S4.** The optimization of lambda exo incubation time in a buffer solution. S-5

**Figure S5**. The optimization of concentrations of SA beads to 200 nM SA3 in a buffer solution. S-6

**Figure S6.** The original flow cytometry data of ALP detection. S-6

**Figure S7.** The detection for ALP spiked in 20% human serum. S-7

**Figure S8.** The detection for ALP spiked in Hela cell lysate. S-7

**Figure S9.** PNPP method for ALP detection. S-8

**Figure S10.** ALP inhibitor screening of L-cycsteine. S-8

**References**  S-9

|  | Sequences |
| --- | --- |
| SA1 | FAM-5’-ATTGACCGCTGTGTGACGCAACACTCAAT-3’ |
| cDNA1 | PO_4_-5’- ATTGAGTGTTGCGTCACACAGCGGTCAAT-3’ |
| SA2 | FAM-5’-TTGACCGCTGTGTGACGCAACACTCAA-3’ |
| cDNA2 | PO_4_-5’- TTGAGTGTTGCGTCACACAGCGGTCAA-3’ |
| SA3 | FAM-5’-ATTGACGCGTGTGACGCAACACTCAAT-3’ |
| cDNA3 | PO_4_-5’-ATTGAGTGTTGCGTCACACGCGTCAAT-3’ |

**Table S1.** DNA sequences list.

| Detection  methods | Limit of Detection (LOD) | The minimum incubation time required for ALP detection | Biological Sample | Reference |
| --- | --- | --- | --- | --- |
| Fluorescence | 0.19 U/L | ~1 h | 1% human serum | 1 |
| Fluorescence | 2.17 U/L | ~1.5 h | 2% human serum | 2 |
| Fluorescence | 60 U/L | ~2 h | Living cells | 3 |
| Fluorescence | 10 U/L | 20 min | Not given | 4 |
| Absorbance | 32 U/L | ~1 h | 1% A549 cell lysate | 5 |
| Fluorescence | 5 U/L | ~2 h | 1% bovine serum | 6 |
| Absorbance | 0.1 U/L | ~4 h | 1% human serum | 7 |
| Fluorescence | 12 U/L | 30 min | 20% human serum and cell lysate | This work |

**Table S2**. Comparison of several recent detection methods for alkaline phosphatase.


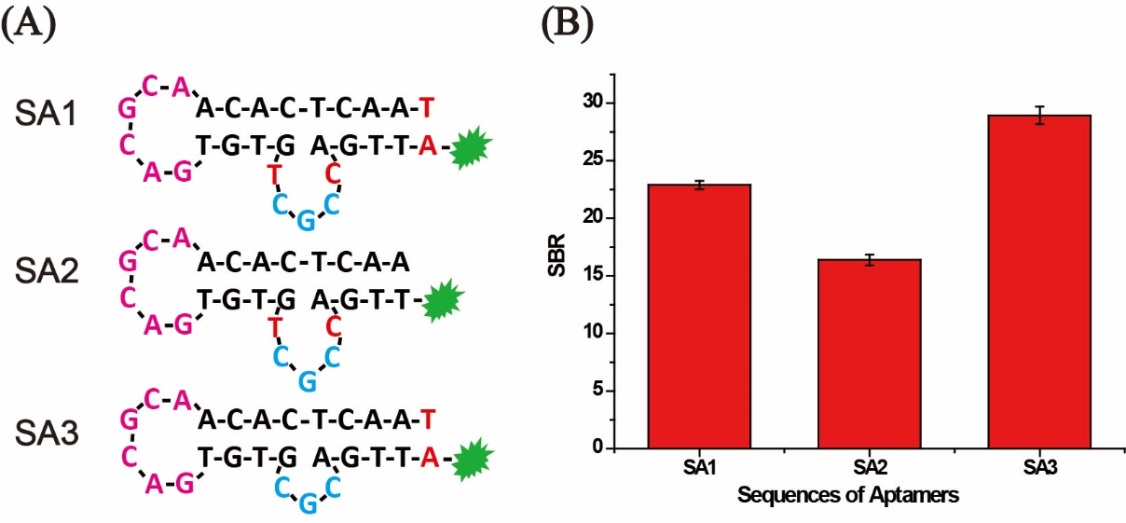


**Figure S1**. The optimization of SA aptamer sequences. (A) Sequences and secondary structures of three designed SA aptamers. Bases in pink, blue and red, respectively, represent the loop, the bulge, and different bases among aptamers. (B) Signal-Background ratio (SBR) of various AP (SA aptamers and their corresponding cDNA) incubated with or without 10 U/mL ALP. SBR was calculated by F_0_/F, where F_0_ and F are fluorescence intensities without and with ALP, respectively. Error bars indicate the standard deviations of three samples.


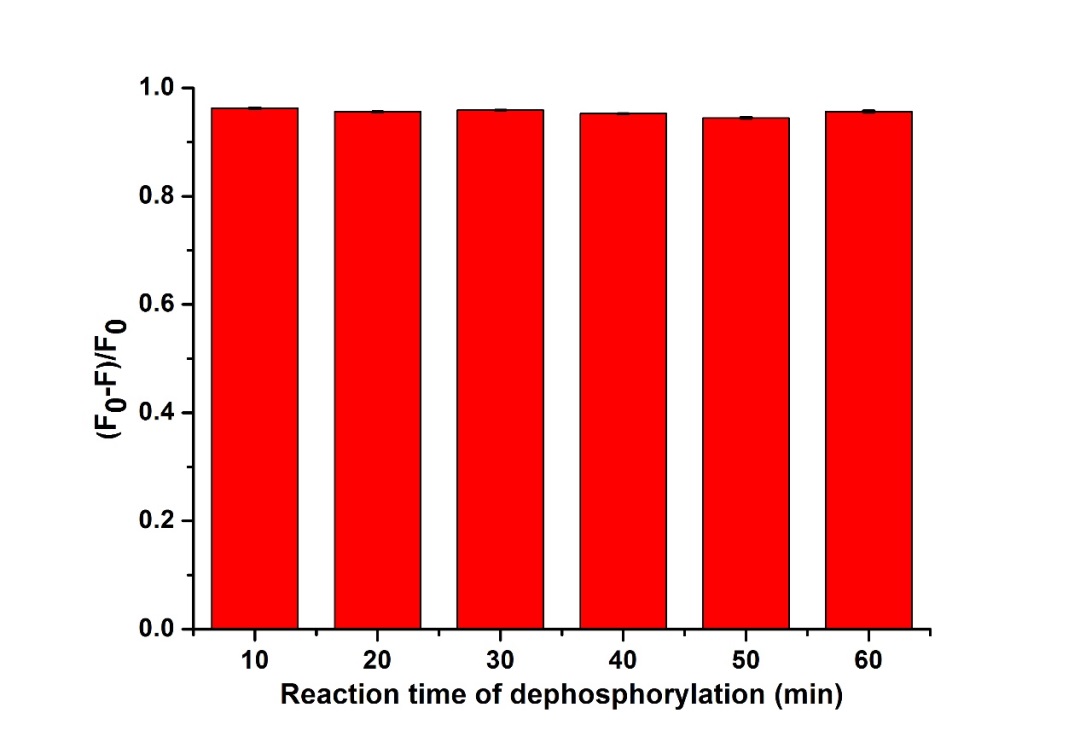


**Figure S2.** Response of AP to ALP with different ALP incubation times in a buffer solution. F_0_ and F are fluorescence intensities without and with ALP, respectively. The concentrations of ALP and λ exo are 10 U/mL and 100 U/mL, respectively. Error bars indicate the standard deviations of three samples.


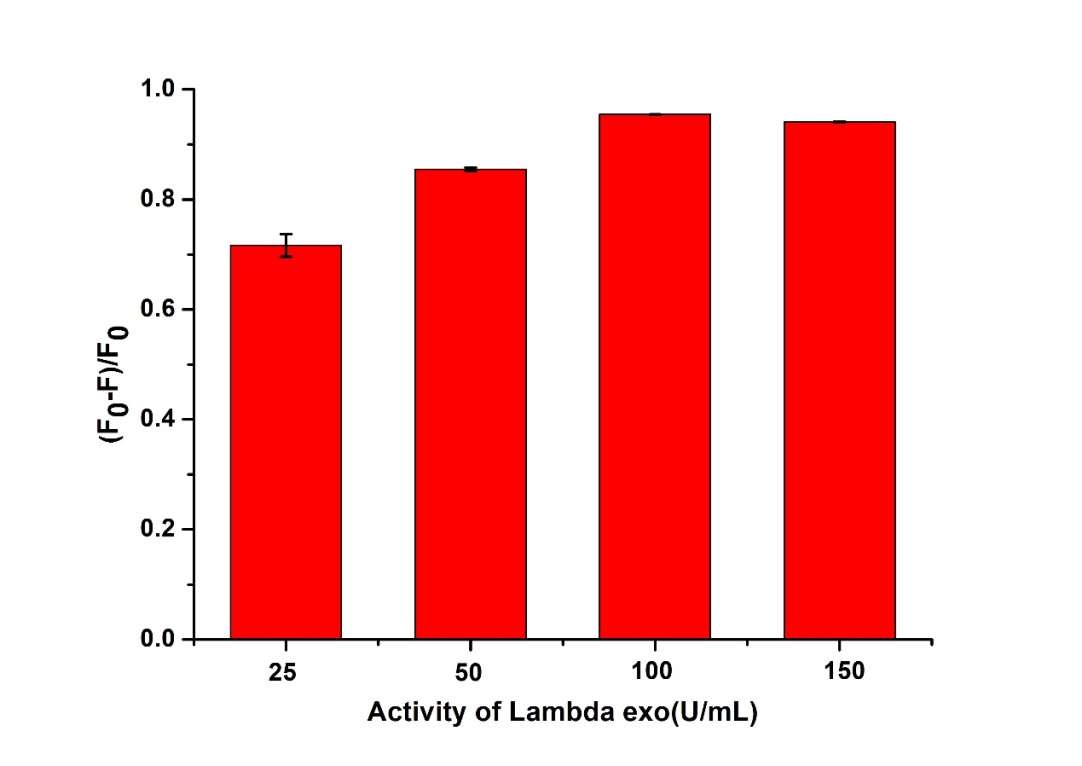


**Figure S3.** Response of AP to ALP with different concentrations of λ exo in a buffer solution. F_0_ and F are fluorescence intensities without and with ALP, respectively. The concentration of ALP is 10 U/mL. Error bars indicate the standard deviations of three samples.


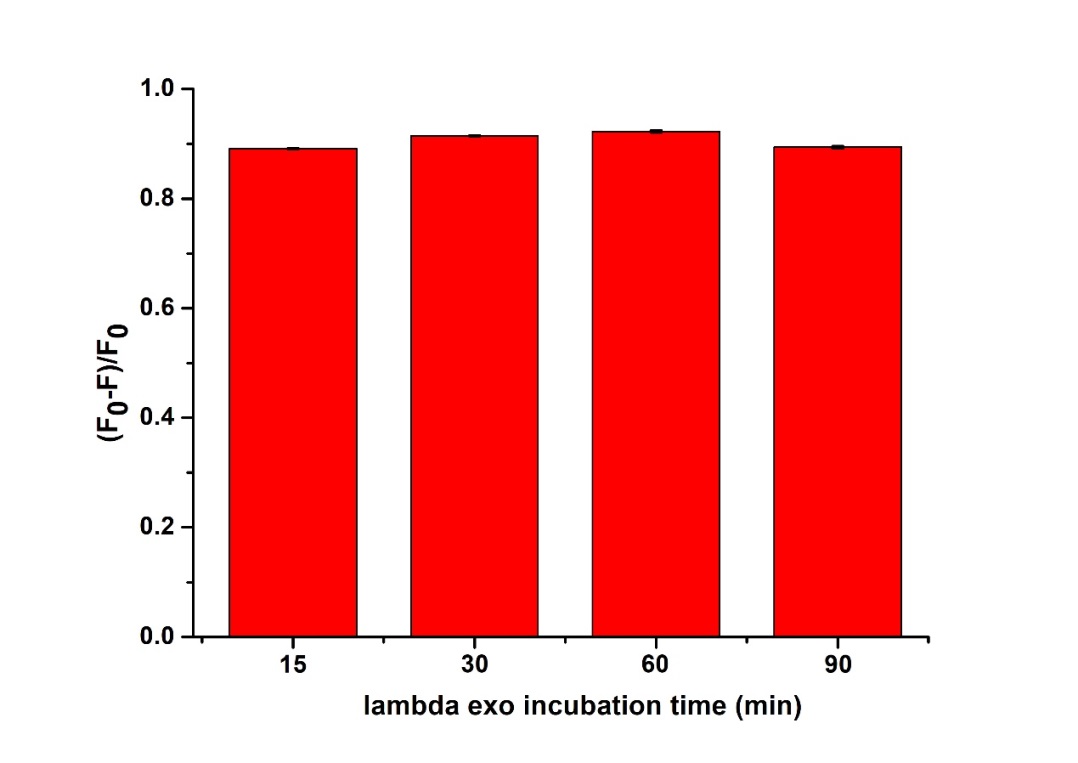


**Figure S4.** Response of AP to ALP with different incubation time of λ exo in a buffer solution. F_0_ and F are fluorescence intensities without and with ALP, respectively. The concentration of ALP is 1 U/mL. Error bars indicate the standard deviations of three samples.


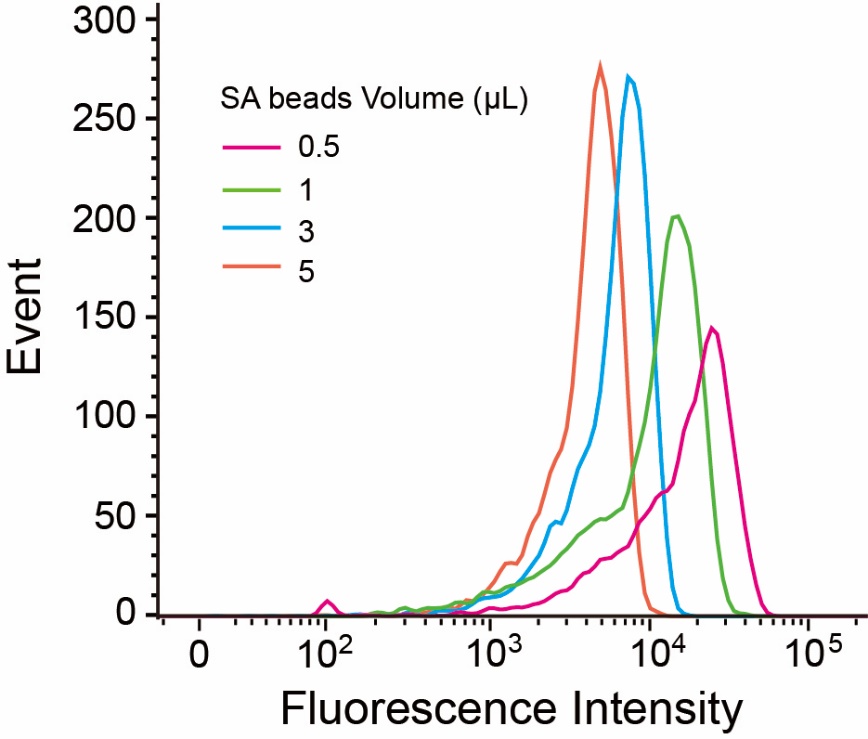


**Figure S5.** Fluorescence Intensity of different concentrations of SA beads for binding to 200 nM SA3 in a buffer solution.


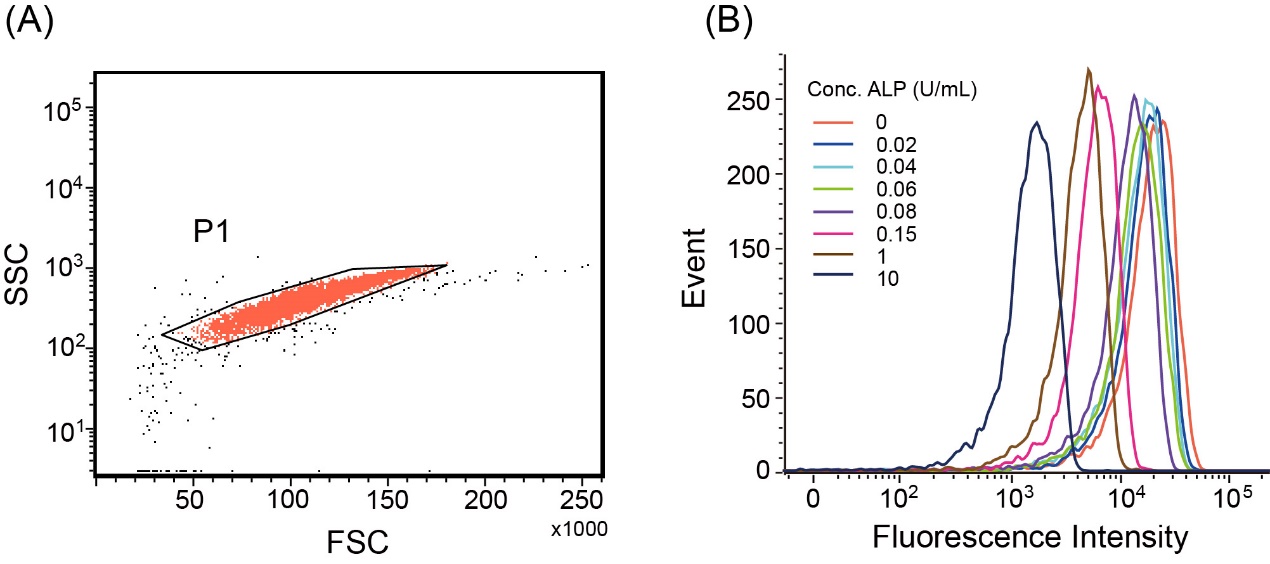


**Figure S6.** (A) A typical scatter diagram of beads. (B) Original flow cytometry data of detection of ALP for plotting Figure 3A.


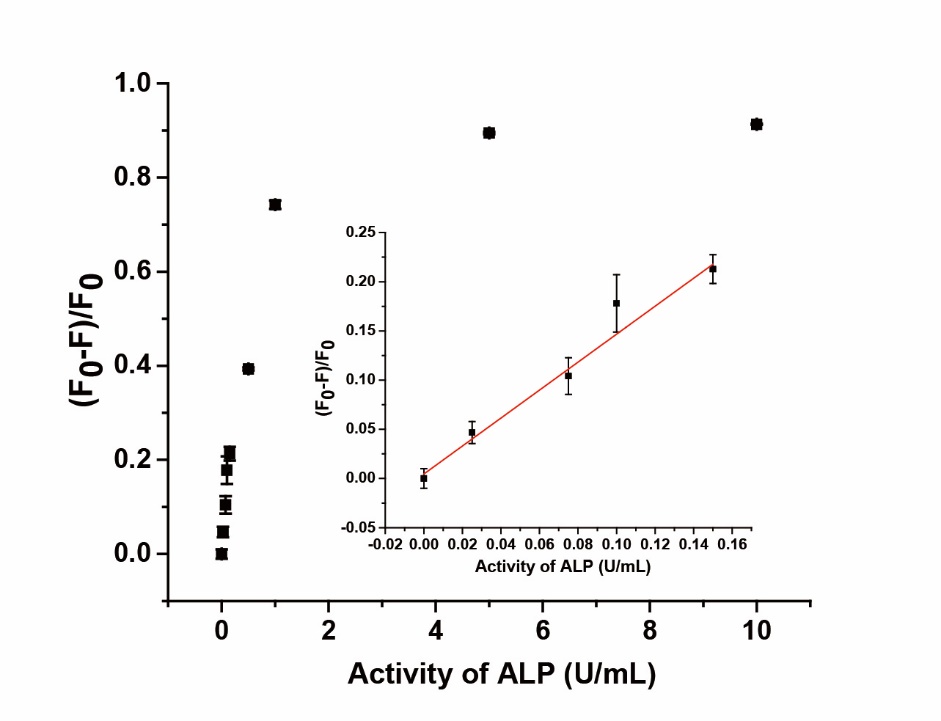


**Figure S7.** Response of quantitative detection of ALP range from 0 to 10 U/mL in 20% human serum monitored by flow cytometry. Inset is the calibration curve range from 0.025 to 0.15 U/mL. F_0_ and F are fluorescence intensities without and with ALP, respectively. Error bars indicate the standard deviations of three samples.


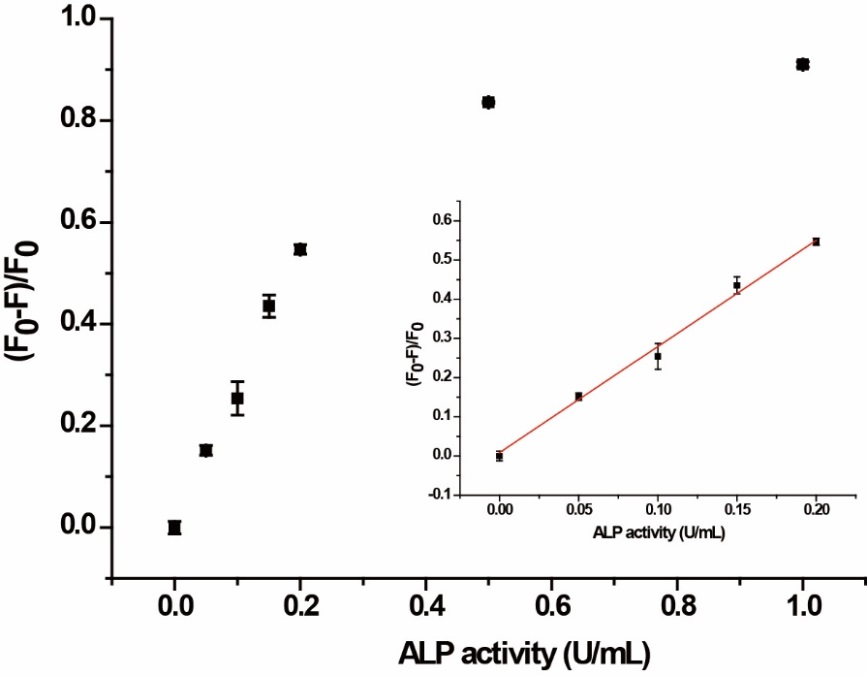


**Figure S8.** Quantitative detection of ALP ranging from 0 to 1 U/mL in spiked Hela cell lysate of 10^5^ cell/mL with AP monitored by flow cytometry. The calibration curve ranges from 0.05 to 0.20 U/mL and the limit of detection is 0.014 U/mL (3σ/S). Error bars indicate the standard deviations of three samples.


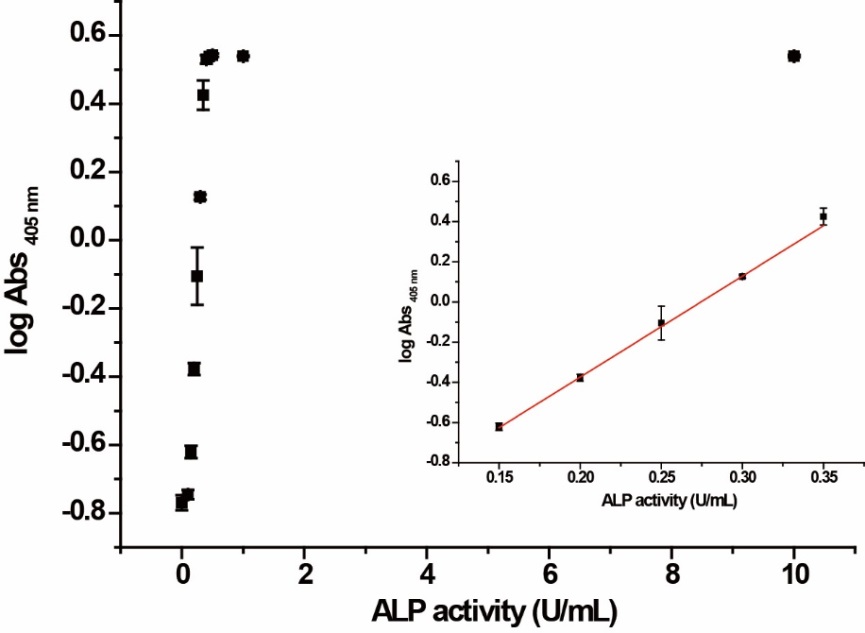


**Figure S9.** Quantitative detection of ALP ranging from 0 to 10 U/mL with PNPP substrate by monitoring the absorption at 405 nm. The calibration curve ranges from 0.15 to 0.35 U/mL and the limit of detection is 0.13 U/mL (3σ/S). Error bars indicate the standard deviations of three samples.

**
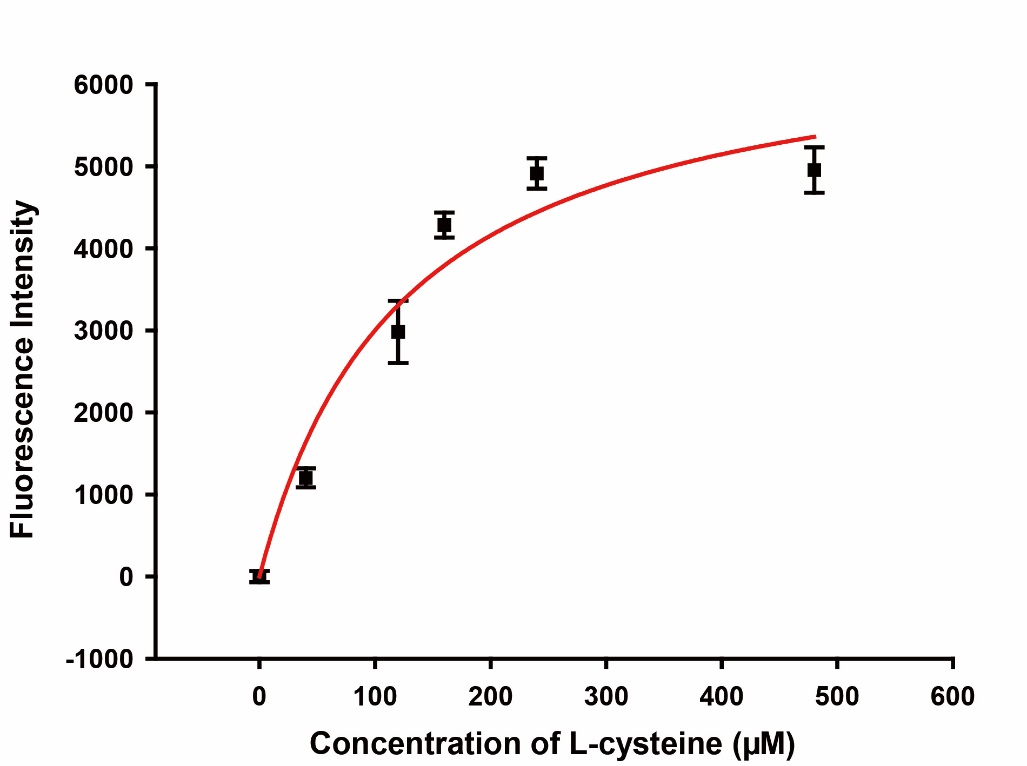
**

**Figure S10.** Response of the AP to 2 U/mL ALP with inhibitor L-cysteine. Error bars indicate the standard deviations of three samples.

**References:**

1. Liu, X.; Xing, X.; Li, B.; Guo, Y.; Zhang, Y.; Yang, Y.; Zhang, L., Fluorescent assay for alkaline phosphatase activity based on graphene oxide integrating with lambda exonuclease. *Biosens. Bioelectron.* **2016**, 81, 460-464.

2. Xing, X.; Liu, X.; Zhou, Y.; Xu, D.; Pang, D.; Tang, H., Graphene oxide enhanced specificity at aptamer and its application to multiplexed enzymatic activity sensing. *RSC. Adv.* **2016**, 6 (14), 11815-11821.

3. Dong, L.; Miao, Q.; Hai, Z.; Yuan, Y.; Liang, G., Enzymatic Hydrogelation-Induced Fluorescence Turn-Off for Sensing Alkaline Phosphatase in Vitro and in Living Cells. *Anal. Chem.* **2015**, 87 (13), 6475-6478.

4. Deng, J.; Yu, P.; Wang, Y.; Mao, L., Real-time Ratiometric Fluorescent Assay for Alkaline Phosphatase Activity with Stimulus Responsive Infinite Coordination Polymer Nanoparticles. *Anal. Chem.* **2015**, 87 (5), 3080-3086.

5. Jiao, H.; Chen, J.; Li, W.; Wang, F.; Zhou, H.; Li, Y.; Yu, C., Nucleic Acid-Regulated Perylene Probe-Induced Gold Nanoparticle Aggregation: A New Strategy for Colorimetric Sensing of Alkaline Phosphatase Activity and Inhibitor Screening. *ACS Appl. Mat. Interfaces* **2014**, 6 (3), 1979-1985.

6. Ma, J.; Yin, B.; Wu, X.; Ye, B., Copper-Mediated DNA-Scaffolded Silver Nanocluster On-Off Switch for Detection of Pyrophosphate and Alkaline Phosphatase. *Anal. Chem.* **2016**, 88 (18), 9219-9225.

7. Hu, Z.; Chen, J.; Li, Y.; Wang, Y.; Zhang, Q.; Hussain, E.; Yang, M.; Shahzad, S. A.; Yu, D.; Yu, C., Nucleic acid-controlled quantum dots aggregation: A label-free fluorescence turn-on strategy for alkaline phosphatase detection. *Talanta* **2017**, 169, 64-69.
